# *Yersinia ruckeri*, the causative agent of enteric redmouth disease in fish

**DOI:** 10.1186/s13567-015-0238-4

**Published:** 2015-09-24

**Authors:** Gokhlesh Kumar, Simon Menanteau-Ledouble, Mona Saleh, Mansour El-Matbouli

**Affiliations:** Clinical Division of Fish Medicine, Department for Farm Animals and Veterinary Public Health, University of Veterinary Medicine, Vienna, Austria

## Abstract

Enteric redmouth disease (ERM) is a serious septicemic bacterial disease of salmonid fish species. It is caused by *Yersinia ruckeri*, a Gram-negative rod-shaped enterobacterium. It has a wide host range, broad geographical distribution, and causes significant economic losses in the fish aquaculture industry. The disease gets its name from the subcutaneous hemorrhages, it can cause at the corners of the mouth and in gums and tongue. Other clinical signs include exophthalmia, darkening of the skin, splenomegaly and inflammation of the lower intestine with accumulation of thick yellow fluid. The bacterium enters the fish via the secondary gill lamellae and from there it spreads to the blood and internal organs. *Y. ruckeri* can be detected by conventional biochemical, serological and molecular methods. Its genome is 3.7 Mb with 3406–3530 coding sequences. Several important virulence factors of *Y. ruckeri* have been discovered, including haemolyin YhlA and metalloprotease Yrp1. Both non-specific and specific immune responses of fish during the course of *Y. ruckeri* infection have been well characterized. Several methods of vaccination have been developed for controlling both biotype 1 and biotype 2 *Y. ruckeri* strains in fish. This review summarizes the current state of knowledge regarding enteric redmouth disease and *Y. ruckeri*: diagnosis, genome, virulence factors, interaction with the host immune responses, and the development of vaccines against this pathogen.

## Table of contents

 1. Introduction

2. Identification and classification

3. Clinical signs and pathology of the disease

4. Distribution of *Yersinia ruckeri*

5. Transmission and epidemiology

6. Route of entry and spread

7. Genome

8. Diagnosis

9. Virulence factors

10. Host immune response

11. Control/treatment

11.1 Antibiotherapy

11.2 Probiotics

11.3 Vaccination

12. Conclusions

13. Abbreviations

14. Competing interests

15. Authors’ contributions

16. Acknowledgements

17. References

## 1. Introduction

Fish constitute a major source of protein, fatty acids, vitamins, minerals and essential micronutrients for an expanding segment of the world population. Consequently, aquaculture is the fastest growing food production sector and accounts for approximately 50% of the fish consumed worldwide [1]. Disease outbreaks have become a major constraint to the expansion of aquaculture and have a significant impact on the economic development of many countries. Enteric redmouth disease (ERM, yersiniosis) is one of the most important diseases of salmonids and leads to significant economic losses [2]. The disease is caused by *Yersinia ruckeri*, a Gram-negative rod-shaped enterobacterium, which was first isolated from rainbow trout (*Oncorhynchus mykiss*) in the Hagerman Valley of Idaho, USA [3] and is currently found throughout North and South America, Europe, Australia, South Africa, the Middle East and China [4,5]. Although infections have been reported in other fish species, rainbow trout are especially susceptible to ERM [2-4]. Rainbow trout are fast growing and robust under farming conditions, and thus are the most widely farmed salmonid fish [1]. Herein, we review the latest scientific developments on *Y. ruckeri*, including the present distribution and diagnosis of ERM, the route of entry of *Y. ruckeri* into the fish, its genome, virulence factors, interactions with the host and immune responses, and methods to control yersiniosis.

## 2. Identification and classification

*Yersinia* is a genus of Gram-negative, rod-shaped, facultative anaerobes within the family *Enterobacteriaceae. Yersinia* comprises several pathogenic species, which cause diseases in humans and other animals, including fish. *Yersinia ruckeri* is the causative agent of enteric redmouth disease in various species of salmonids worldwide. It was described from rainbow trout in the Hagerman Valley of Idaho, USA in the 1950s [3]. The *Y. ruckeri* bacillus is approximately 0.75 μm in diameter and 1–3 μm in length. *Y. ruckeri* has a 3.7Mb genome, with a ~47% G + C ratio [6,7], the same as other *Yersinia* species [7,8]. High-throughput DNA sequencing of *Yersinia* species has confirmed that *Y. ruckeri* shares the same core set of genes with the other members of the genus [9].

Different strains of *Y. ruckeri* have been reported and categorized on the basis of serotypes, biotypes and outer-membrane protein types. In 1993, the typing scheme was updated and species were further subdivided into four serotypes with different subgroups: Serotype O1 is subdivided into two subgroups O1a (serovar I) and O1b (serovar III) and serotype O2 (serovar II) into three subgroups O2a, O2b and O2c. The remaining serotypes are designated as serotype O3 (serovar V) and serotype O4 (serovar VI) [10]. The vast majority of epizootics in salmonids is caused by motile serotype O1a [10].

Strains of *Y. ruckeri* have also been subdivided into two biotypes. Strains of biotype 1 are positive for motility and lipase secretion, whereas strains of biotype 2 are negative for both tests [2,4]. However, the ability to secrete lipase appears to have little relevance to the virulence of *Y. ruckeri* during the natural infection [11]. *Y. ruckeri* is characterized biochemically as glucose-fermentative, catalase-positive, nitrate-reductive, oxidase-negative, with the ability to secrete b-galactosidase, lysine and ornithine decarboxylases but neither hydrogen sulfide nor indole [2,4].

The genetic structure and variations within *Y. ruckeri* have been investigated using molecular tools including multilocus enzyme electrophoresis, pulsed-field gel electrophoresis (PFGE), fatty acid methyl ester profiles, ribotypes and interspersed repetitive sequences-PCR. These have shown that O1a strains of *Y. ruckeri* have high levels of genetic homogeneity [12,13]. Bastardo et al. [14] investigated the diversity and evolutionary relationships among a geographically and temporally diverse collection of *Y. ruckeri* strains using a multilocus sequence typing scheme. These authors suggested the existence of two major clonal complexes (CC1 and CC2) within the *Y. ruckeri* population structure. They support the ‘epidemic’ model of clonal expansion, in which populations of well-adapted clones explode to be widely distributed. Genetic and antigenic differences have been found between biotype 1 and biotype 2 strains, using 16S rRNA sequence analysis, genotyping (including ERIC-PCR and (GTG)_5_-PCR), and Western blot analysis [15,16]. Furthermore, Welch [17] developed a novel PCR-based assay to detect mutant alleles in strains of *Y. ruckeri* using *fliR* gene primers, restriction enzyme digestion and sequencing of the resulting fragments. This assay identified four mutant alleles in biotype 2 strains of *Y. ruckeri* that are presently circulating in Europe and the United States, and which can cause outbreaks in vaccinated fish.

## 3. Clinical signs and pathology of the disease

**Figure 1:**
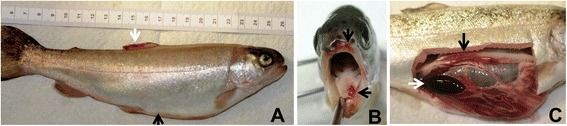
**Rainbow trout showing clinical signs of enteric redmouth disease. A**: darkening of the skin, *enlarged abdominal valley* (*black arrow*), and hemorrhages in the dorsal fin (*white arrow*). **B**: hemorrhages in and around the mouth (*arrows*). **C**: enlarged and black spleen (*white arrow*), and reddened intestine (*black arrow*).

ERM can affect fish from all age classes but it is most acute in young fish (fry and fingerlings). The disease appears as a more chronic condition in older/larger fish. Disease outbreaks start with low level mortalities that are sustained over time, resulting in high cumulative stock losses [2,4]. Changes in fish behavior may be observed, including swimming near the surface, lethargic movements and loss of appetite. Other signs of disease include exophthalmia and darkening of the skin, and subcutaneous hemorrhages in and around the mouth and throat, which give the disease its common name. Petechial hemorrhages may occur on the surfaces of the liver, pancreas, pyloric caeca, swim bladder and in the lateral muscles. The spleen is often enlarged and can be almost black in color (Figure 1), and the lower intestine can become reddened and filled with an opaque, yellowish fluid [2,4].

**Figure 2:**
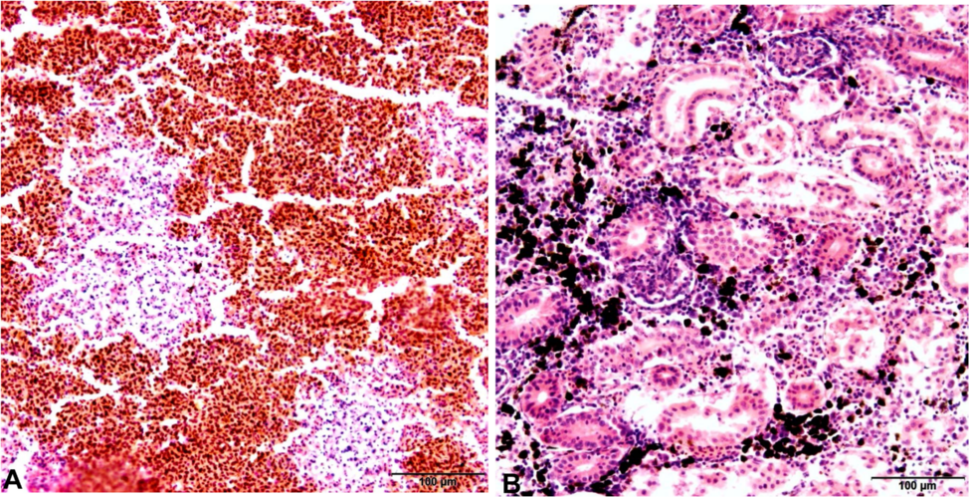
**Histological sections of spleen and kidney organs of rainbow trout infected with** ***Y. ruckeri*** **. A**: multifocal necrosis can be seen in the spleen. **B**: degeneration of interstitial tissue and a marked increase in melano-macrophages can be seen in the kidney. Sections were stained with haematoxylin and eosin (H&E).

Histopathological examination shows general septicaemia with inflammation in most organs, and particularly kidney, spleen, liver, heart, gills and in areas with petechial haemorrhage. Pathological changes in the gills, including hyperemia, oedema and desquamation of the epithelial cells in the secondary lamellae have been described [2,4,18]. Focal areas of necrosis can be present in the spleen (Figure 2A), kidney (Figure 2B) and liver. In the kidney, degenerated renal tubules, glomerular nephritis and a marked increase in melano-macrophages may be observed [2,4,18].

## 4. Distribution of *Y. ruckeri*

Since the first report of *Y. ruckeri* infection in rainbow trout in the USA [3], the pathogen has been isolated from multiple other fish species worldwide, including Canada, Europe, South America, the Middle East, China, India and Australia [4,5,19]. Additionally, *Y. ruckeri* has been isolated from animals other than fish, including muskrat (*Ondatra zibethicus*), kestrel (*Falco* spp.), sea gulls (*Laridae*), turtles (*Cheloniidae*) and humans [4,20,21]. These numerous reports demonstrate that *Y. ruckeri* has a wide host range and geographical distribution, and can cause both epizootics and zoonosis.

## 5. Transmission and epidemiology

*Y. ruckeri* infections can be transmitted by direct contact between infected and non-infected fish. A carrier state for *Y. ruckeri* was demonstrated, where infectious fish can survive 2 months after both experimental [4] and natural infections [4,22]. Busch and Lingg [23] verified that up to 25% of a rainbow trout population could carry *Y. ruckeri* in the lower intestine. The bacteria can then be released when the carrier fish become stressed. For example, it was observed that carriers transmitted *Y. ruckeri* to clinically healthy fish when the temperature was raised to 25 °C, but no transmission occurred from unstressed carrier fish [24]. Shedding of the bacterium in the feces is also likely to play an important role in transmission, and *Y. ruckeri* can survive at least 4 months outside the host [23]. The bacterial cells use either pili or flagella to move along surfaces to link with other bacteria and form or enlarge microcolonies [25,26]. The over-expression of flagellar proteins is a phenotypic characteristic of bacteria associated with high adhesiveness and is essential to initiate development of biofilms. It is now well recognized that the formation of biofilms is an important feature of the survival of bacteria on surfaces and in sediments in aquatic environments [26,27]. Coquet et al. [27] isolated a *Y. ruckeri* strain that was able to form biofilms on the solid supports frequently found in fish farm tanks. These biofilms are reported to be a source of recurrent infection in rainbow trout facilities [4,27]. The spread of *Y. ruckeri* has also been linked to putative vectors, which include aquatic invertebrates and birds [20]. While vertical transmission from mother to progeny has not been well studied, *Y. ruckeri* has been recovered from disinfected non-fertilized eggs of Chinook salmon (*Oncorhynchus tshawytscha*), whose offspring experienced low mortalities from fertilization to 12 weeks on feed [28]. Furthermore, the recent discovery of *Y. ruckeri* DNA in unfertilized eggs and ovarian fluid of Chinook salmon suggests that the pathogen could be transmitted vertically. However, this study was unable to verify the occurrence of bacterial cells within the chorion of the egg [28].

## 6. Route of entry and spread

Histological examination of rainbow trout experimentally infected with *Y. ruckeri* indicated that gills are an important portal of entry for *Y. ruckeri.* Thereafter, *Y. ruckeri* spreads to the other organs [18]. This spread was recently visualized in organs of rainbow trout using optical projection tomography and immunohistochemistry [29]. The authors suggested that *Y. ruckeri* initially infects the secondary gill lamellae, then spreads to the blood system via the gill pavement cells, as rapidly as 1 min post infection (mpi). It could be detected in the lumen of the intestine at 30 mpi, in the kidney at 3 days post infection (dpi), and in the liver, spleen, brain and heart at 7 dpi. *Y. ruckeri* was no longer detectable in the liver, spleen, brain and heart at 21 dpi [29].

## 7. Genome

Annotated whole genome sequences of two strains of *Y. ruckeri* are currently available: the motile CSF007-82 strain, isolated from diseased rainbow trout and the motile O1b 37551 strain, isolated from vaccinated Atlantic salmon (*Salmo salar*) (GenBank accession numbers PRJEB6967 and JPFO00000000, respectively) [7,30]. The genome of CSF007-82 is 3 799 036 bp and contains 3530 coding sequences (CDS), 80 tRNA and 7 ribosomal operons, while that of O1b 37551 is 3 775 486 bp and contains 3,406 CDS, 56 tRNA and 4 rRNA genes [7,30]. The availability of these whole genomes is contributing to better understanding of pathogenesis and virulence factors, facilitating identification of mutations and construction of new targets for vaccines. However, whole genome sequences of non-motile *Y. ruckeri* strains from different geographical locations are still needed to gain insight into the differences between motile and non-motile strains.

## 8. Diagnosis

**Figure 3:**
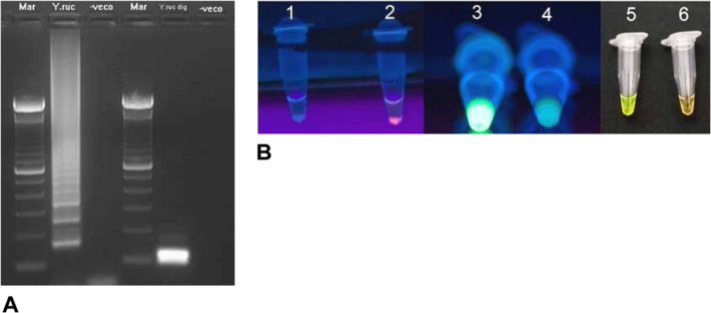
**Detection methods for** ***Yersinia ruckeri*** **using loop-mediated isothermal amplification method. A**: Agarose gel showing ERM-LAMP products of *Y. ruckeri*; Lane mar: 100 bp DNA ladder, lane Y. ruc: amplified *Y. ruckeri* LAMP product, lane Y. ruc dig: Hph I digested *Y. ruckeri* LAMP products of 87 and 108 bp, and lane veco: negative control. **B**: Visual detection of ERM LAMP products using SYBR Green I stain 1: 1: Negative control reaction using Rox- labelled probe, there is neither pellet nor red fluorescence, 2: positive control reaction using Rox- labelled probe, the pellet emitted red fluorescence; 3: positive sample by using FDR, emitted strong green fluorescence when exposed to UV light; 4: negative sample by using FDR, did not emit strong green fluorescence under UV light; 5: positive sample with green color using SYBR green I stain; 6: negative sample with orange color using SYBR green I stain (Image from Saleh et al. [35] with permission).

Multiple diagnostic assays have been developed for *Y. ruckeri*, including culturing, serological tests and molecular biological techniques [4]. *Y. ruckeri* has been isolated using Tryptic soy agar, Columbia blood agar and MacConkey agar [2,4,31,32]. While *Y. ruckeri* can grow at a wide range of temperatures, its thermal optimum is 20–28 °C [2,4]. *Y. ruckeri* can be detected using ELISA, agglutination and immunofluorescence antibodies [33]. Molecular detection techniques include restriction fragment length polymorphism [34], loop-mediated isothermal amplification (LAMP) [35] and polymerase chain reaction (PCR) [32,36,37]. PCR-based amplification of the 16S rRNA gene can detect *Y. ruckeri* in tissues of infected fish [32,36,37]. A LAMP assay was optimized by Saleh et al. [35] and amplifies the yruI/yruR gene, which encodes the *Y. ruckeri* quorum sensing system. This ERM-LAMP assay is sensitive, rapid and the amplification products can be detected by visual inspection or gel electrophoresis (Figure 3).

The PCR method described by Gibello et al. [32] has the advantage of being able to detect low levels of *Y. ruckeri*, and thus provide the possibility to detect asymptomatic carriers; essential to avoid transmission and spread of ERM. PCR detection of *Y. ruckeri* in the blood of rainbow trout was described by Altinok et al. [37]. The use of blood samples is a non-lethal sampling method and permits repetitive sampling of individual fish. Other nonlethal methods include culture of feces and biopsy of head kidney by dorsocaudal aspiration of the kidney tissue [38]. Improvements in specificity of both conventional and quantitative PCR (qPCR) assay have been attained using other candidate genes [39-41]. Bastardo et al. [42] recently described a qPCR method based on the recombinant protein A (recA) gene*.* Gold nanoparticle-based assays are an emerging technology, which may become a powerful tool for rapid, direct and sensitive detection of unamplified *Y. ruckeri* nucleic acids in clinical samples. In nanoparticle-based assays, DNA or RNA hybridizes with pathogen-specific probes that are attached to the surface of the gold nanoparticles. Hybridization occurs at a determined temperature and time, and results in aggregation of the nanoparticles and a concomitant red to blue color change [43].

## 9. Virulence factors

Several virulence mechanisms of *Y. ruckeri* have been identified, and found to differ with the geographical origin of the isolate. Several extra-cellular products (ECP) have been shown to reproduce the clinical signs associated with the hemorrhagic form of the disease when injected into their host [10]. Multiple molecules are known to contribute to the virulence of these ECP, for example: the iron-regulated *Serratia*-like haemolysin YhlA, which has cytolytic and haemolytic activity [44], an azocasein protease [45], and the 47 kDa metalloprotease Yrp1, which has a wide range of targets and is particularly efficacious at degrading fibronectin, actin and myosin [46,47]. Recently, *yrpA* and *yrpB* have been demonstrated to be induced in the gut of rainbow trout [48]. In addition, genes involved in the catecholate siderophore ruckerbactin iron acquisition system were shown to be over-expressed by the bacterium during the infection process in fish. Inactivation of the corresponding gene led to a hundred-fold increase in the LD_50_ of the bacterium [49]. Heat sensitive factor (HSF) produced by the alkylsulphatase enzyme YraS has been proposed as a virulence factor [50], and a differential culture medium, using Coomassie Brilliant Blue has been developed to identify strains that carry this factor [51]. However, recent findings have contradicted these results and suggested that HSF might not be required for *Y. ruckeri* virulence [52]. Similarly, the cdsAB operon was initially identified through in vitro expression technology [49] and has been shown to be required for uptake of L-cysteine by the bacterium [53]. However, despite L-cysteine being present in fish serum, mutations in the cdsAB operon failed to impact the growth of *Y. ruckeri*, raising questions about the necessity of the cdsAB operon [54].

Finally, as is often the case in microbial pathogens, the expression of these virulence factors appears tightly regulated and linked with both the availability of iron [44] and the concentration of auto-inducing molecules [41,55]. BarA-UvrY is a regulator identified in *Y. ruckeri* [55] and has homologs in other enterobacteria. BarA-UvrY is involved in resistance to oxidative killing, and the invasion of host cells. However, on the contrary to observations in other enterobacteria, mutations in BarA-UvrY did not impact the growth of *Y. ruckeri* under iron-limited conditions [56]. More research is still needed to identify novel virulence genes and to understand the full pathogenic mechanisms of *Y. ruckeri* during the infection process in the fish.

## 10. Host immune response

Non-specific and specific immune responses of fish against *Y. ruckeri* strains have been studied extensively. For example, both O-antigen and formalin-inactivated *Y. ruckeri* cells induced an immune response in rainbow trout [57,58], producing peak levels of antibody in the spleen at 14 days post exposure (dpe) and overall maximum titer at 28 dpe [57]. Similarly, intra-peritoneal booster doses of *Y. ruckeri* bacterin induced a significant immune response in rainbow trout at 146 days post injection [58]. Phagocytic cells, such as neutrophils and macrophages, are an important part of the fish host immune system, and an inflammatory response to *Y. ruckeri* has been observed in the body cavity of rainbow trout [59]. Gene expression levels of CXCd, cytokine, chemokine, interleukin, cell receptor, immunoglobulin, SCOS and CISH genes have been measured in rainbow trout in response to *Y. ruckeri* biotype 1 strains [60-64] and *Y. ruckeri* biotype 2 strains using quantitative real-time PCR [65,66]. Recent discoveries suggest production of specific antibodies against *Y. ruckeri* may play a role in protection against disease [63]. Serum amyloid A, which belongs to a highly conserved group of apolipoproteins, is considered to be an important innate immune molecule in rainbow trout during the course of *Y. ruckeri* infection [67]. All these studies contribute to our understanding of how the innate and adaptive immune systems in rainbow trout respond to both primary infection (first infection) and re-infection (secondary infection). It is worthy of note that all of these studies were based on mRNA expression, which may not always accurately reflect protein expression and biochemical changes [68]. Mechanisms of biochemical changes in the organs of fish infected with strains of *Y. ruckeri* biotypes 1 and 2 at the proteomics level still need to be investigated, both to understand the proteomic background for observed proteomic changes, and to elucidate the mechanism of action of the proteins whose expression differs between the biotypes.

## 11. Control/treatment

### 11.1 Antibiotherapy

As is often the case with fish bacterial pathogens, post-infection treatment relies mostly on the use of antibiotics. Unfortunately, only a limited spectrum of compounds is routinely used [69]: amoxicillin; oxolonic acid; oxytetracycline; sulphadiazine in combination with trimethroprim and, more recently, florfenicol [70]. This narrow range of options may facilitate the emergence of antibiotic resistance [71]. Screening of *Y. ruckeri* isolates has shown that only 1 out of 50 was resistant to florfenicol [70]. Similarly, a more recent review suggested that most European isolates are still widely responsive to antibiotherapy [72]. While a β-lactamase gene has been discovered on the chromosome of *Y. ruckeri* [73], there is evidence that this gene is not likely to be expressed at high levels [74]. In vitro testing has, however, shown that *Y. ruckeri* readily develops resistance against oxolinic acid, oxytetracycline and potentiated sulphonamide [75]. Finally, and more anecdotally, *Y. ruckeri* is both a natural producer of and naturally resistant to the antibiotic holomycin [76].

### 11.2 Probiotics

Concern about the development of antibiotic resistance has spurred research into alternative methods for controlling bacteria, in particular, beneficial probiotic bacteria and yeast [77]. Some success has been demonstrated for the use of probiotics to fight *Y. ruckeri*: oral administration of *Bacillus subtilis* and *Bacillus licheniformis* protects rainbow trout against subsequent infections [78]. The authors hypothesized that this protection was due to either anti-microbial secretions by the bacterium or to its immuno-stimulating effect. This later hypothesis was supported by data that showed that injection of cell wall components of probiotic strains of *B. subtilis* and outer membrane components including LPS of *Aeromonas sobria*, were also protective [79].

Dietary supplementation with *Bacillus* sp., and *Aeromonas sobria*, was confirmed as protective by Brunt et al. [80]: it reduced mortalities from 80% in the control to 0% (*Bacillus* sp. treatment) and 6% (*A. sobria* treatment). Similarly, *Enterobacter cloacae* fed alongside *Bacillus mojavensis* was shown to reduce mortalities from *Y. ruckeri* challenge from 65to 0.8% [81].

Feed supplementation with *Carnobacterium maltaromaticum* B26 and *Carnobacterium divergens* isolated from the normal intestinal microbiota of rainbow trout were found to be protective against further *Y. ruckeri* infections [82]. Interestingly, studies by Robertson et al. [83] confirmed the protective action of *Carnobacterium* sp. but found no antagonistic activity in vivo, suggesting that, as seems to be the case for *B. subtilis*, protection is achieved by stimulation of host defense rather than direct anti-microbial effect.

In addition, *Lactobacillus lactis* was shown to be antagonistic to *Y. ruckeri* and, as with *Lactobacillus fermentum*, significantly reduces adhesion of the pathogen to fish mucus [84]. Comparable results were reported by Sica et al. [85] who screened 12 lactic acid bacteria and found that 60% of them displayed competitive exclusion against *Y. ruckeri*.

Recently, it was reported that feeding fish with a plant-based diet modified the composition of their gut microflora, and affected their immune response to *Y. ruckeri* [86], suggesting a prebiotic effect. This however did not correlate to a significant difference in mortalities during that trial.

Jaafar et al. [87] investigated the effect of two supplements: organic acids and a combination of β-glucan alongside mannan-oligosaccharides, nucleotides, lactic acid bacteria, and vitamins C and E. The supplements were tested separately and in conjunction, but were found not to have a significant effect on survival of fish exposed to *Y. ruckeri*.

### 11.3 Vaccination

The significant economic losses in salmonid fish aquaculture can be controlled to some extent by use of vaccinations. ERM was one of the first fish diseases for which an effective commercial vaccine was developed [2,88]. The vaccine utilizes monovalent, inactivated whole cell suspensions of *Y. ruckeri* serotype O1 biotype 1, which can be administered to fish by several routes, e.g. immersion, injection and oral. It provides good levels of protection against *Y. ruckeri* biotype 1 strains [89–93], as summarized in Table 1. New vaccines have been developed that are based on the *Y. ruckeri* Yrp1 protease, aroA gene, extracellular product and lipopolysaccharide and these provide good protection against *Y. ruckeri* biotype 1 strains [94-96]. However, *Y. ruckeri* biotype 2 strains are harder to combat and have been responsible for disease outbreaks in fish that had been vaccinated against biotype 1 strains; thus monovalent vaccines fail to induce protection against biotype 2 infection [15,31]. A bivalent vaccine was developed using formalin inactivated biotypes 1 and biotype 2 *Y. ruckeri* strains, and provides good protection against the biotype 2 strains [16,97]. cDNA microarray analyses of gill tissues from unvaccinated and vaccinated Atlantic salmon (*Salmo salar*) challenged with *Y. ruckeri* O1b biotype 1, have led to detection of genes and biosignatures which may be useful as predictive indicators of vaccine success. These targets include: non-protective/pathological response genes (cathelicidin, C-type lection and collagenase), vaccine-induced protective genes (immunoglobulin heavy chain, selenoprotein, 60S ribosomal protein L37 and unknown) and transcriptional biosignature of predominantly immune-relevant genes including hepcidin, immunoglobulin mu heavy chain, mylelin and lymphocyte protein. Detection of these bioindicators demonstrates that there is a range of potential targets for future vaccine development [98].

**Table 1:** **Experimental vaccine trials using a variety of antigen-preparation methods and their protection in fish following experimental infection**

Antigens	Routes	Fish species	Challenge strains	RPS (%)	References
Formalin inactivated high pH *Y. ruckeri* O1, strain Y-11	Immersion	Rainbow trout	*Y. ruckeri* O1, strain Y-12	83–96	[89]
Yrp1 protease toxoid of *Y. ruckeri*, strain 150RI4	i.p.	Rainbow trout	*Y. ruckeri*, strain 150	79	[46]
Live attenuated *Y. ruckeri* O1, strain 21102	i.p.	Rainbow trout	*Y. ruckeri* O1, strain 21102	90	[94]
Formalin inactivated *Y. ruckeri* O1, biotype 1	Bath	Rainbow trout	*Y. ruckeri* O1, strain 392/2003	75–76.9	[61]
Extracellular product of *Y. ruckeri*	Immersion	Rainbow trout	*Y. ruckeri*	74–81.4	[95]
Formalin inactivated *Y. ruckeri* O1b biotype 1 (Yersinivac-B)	Immersion	Atlantic salmon	*Y. ruckeri* O1b, strain TCFB 2282	37	[91]
Trypsinated Yersinivac-B	Immersion	Atlantic salmon	*Y. ruckeri* O1b, strain TCFB 2282	55.6	[91]
Formalin inactivated *Y. ruckeri* serotype O1, biotype 1 × biotype 2 (EX5)	Immersion	Rainbow trout	*Y. ruckeri* O1, strains EX5, 58669, G1S1, DenA, BAS2A	87–100	[16]
Formalin inactivated *Y. ruckeri* serotype O1, biotype 1 × biotype 2 (EX5)	Immersion and i.p.	Rainbow trout	*Y. ruckeri* O1, biotype 2, strain 100415-1/4	100	[97]
Formalin inactivated *Y. ruckeri* biotype 1, strain Hagerman	Immersion	Rainbow trout	*Y. ruckeri* O1, biotype 2, strain 100415-1/4	29.5	[64]
Recombinant flagellin protein of *Y. ruckeri* biotype 1 BA19	i.p.	Rainbow trout	*Y. ruckeri* biotype 1 YR1 and biotype 2 R1	68–72	[99]
Formalin inactivated *Y. ruckeri* biotype 1, strain KC291153	Immersion with montanide adjuvant	Rainbow trout	*Y. ruckeri* biotype 1, strain KC291153	93.8–100	[82]
Lipopolysaccharide of *Y. ruckeri*	i.p.	Rainbow trout	*Y. ruckeri*	77.4–85.1	[94]
Formalin inactivated *Y. ruckeri* biotype 1, strain Hagerman	Oral and anal	Rainbow trout	*Y. ruckeri* O1, biotype 1, strain 392	100	[93]

*i.pc* intraperitoneal injection, *RPS* relative percentage survival.

## 12. Conclusions

*Y. ruckeri* causes significant economic losses, particularly in salmonid aquaculture. Two whole genome sequences of motile strains of *Y. ruckeri* have been annotated and can now be used for comparative genomic analysis of *Y. ruckeri* strains, investigation of gene-level pathogenicity, development of potential drug targets and vaccines. Quantitative proteomic analysis of multiple geographic isolates of biotypes 1 and 2 *Y. ruckeri* strains are not yet completed, and are required to create a proteomic map and understand proteomic changes and differences between the biotypes and strains. Some potential virulence factors of *Y. ruckeri* have been identified but more research on the bacterium’s virulence mechanisms is needed to understand the full pathogenicity of *Y. ruckeri* during the course of infection. Investigations have revealed aspects of the fish immune response to *Y. ruckeri* infections, however there is still an urgent need to improve our understanding of the biochemical changes that occur in host tissues and organs during infection. Biotype 2 strains of *Y. ruckeri* have been responsible for outbreaks in rainbow trout that had been vaccinated against biotype 1, thereby confirming the failure of monovalent vaccines to protect the fish against infection. Formalin inactivated bivalent vaccines can significantly reduce mortalities due to infections with biotype 2 strains but the development of more efficient vaccines against both biotypes of *Y. ruckeri* is still needed.

## 13. Abbreviations

cDNA: Complementary DNA
CDS: Coding sequences
CISH: Cytokine-inducible SH2-containing protein
dpe: Days post exposure
ECP: Extra-cellular product
ERIC-PCR: Enterobacterial repetitive intergenic consensus sequence-based PCR
ERM: Enteric redmouth, HSF: Heat sensitive factor
LAMP: Loop-mediated isothermal amplification
MLST: Multilocus sequence typing
mpi: Minute post infection
PCR: Polymerase chain reaction
PFGE: Pulsed-field gel electrophoresis
qPCR: Quantitative PCR
SCOS: Suppressor of cytokine signaling
*Y. ruckeri*: *Yersinia ruckeri*
